# Characterization, immunostimulatory and antitumor activities of a β-galactoglucofurannan from cultivated *Sanghuangporus vaninii* under forest

**DOI:** 10.3389/fnut.2022.1058131

**Published:** 2022-12-22

**Authors:** Junwen Cheng, Yanbin Wang, Jiling Song, Yu Liu, Weiwei Ji, Liang He, Hailong Wei, Chuanjiu Hu, Yihan Jiang, Yiqi Xing, Xubo Huang, Hongmei Ding, Qinghai He

**Affiliations:** ^1^Key Laboratory of Biological and Chemical Utilization of Zhejiang Forest Resources, Department of Forest Foods, Zhejiang Academy of Forestry, Hangzhou, China; ^2^Institute of Horticulture, Hangzhou Academy of Agricultural Sciences, Hangzhou, China; ^3^Institute of Biochemistry, College of Life Sciences, Institute of Biochemistry, Zhejiang University, Hangzhou, China; ^4^Huzhou Liangxi Forest Park Management Office, Huzhou, China; ^5^School of Forestry and Biotechnology, Zhejiang A & F University, Hangzhou, China; ^6^Center of Forecasting and Analysis, Zhejiang Chinese Medical University, Hangzhou, China

**Keywords:** *Sanghuangporus vaninii*, polysaccharide, structure, antitumor activity, conformation

## Abstract

A biomacromolecule, named as β-galactoglucofurannan (SVPS2), was isolated from the cultivated parts of *Sanghuangporus vaninii* under the forest. Its primary and advanced structure was analyzed by a series of techniques including GC-MS, methylation, NMR, MALS as well as AFM. The results indicated that SVPS2 was a kind of 1, 5-linked β-Glucofurannan consisting of β-glucose, β-galactose and α-fucose with 23.4 KDa. It exhibited a single-stranded chain with an average height of 0.72 nm in saline solution. The immunostimulation test indicated SVPS2 could facilitate the initiation of the immune reaction and promote the secretion of cytokines *in vitro*. Moreover, SVPS2 could mediate the apoptosis of HT-29 cells by blocking them in S phase. Western blot assay revealed an upregulation of Bax, Cytochrome c and cleaved caspase-3 by SVPS2, accompanied by a downregulation of Bcl-2. These results collectively demonstrate that antitumor mechanism of SVPS2 may be associated with enhancing immune response and inducing apoptosis of tumor cells *in vitro*. Therefore, SVPS2 might be utilized as a promising therapeutic agent against colon cancer and functional food with immunomodulatory activity.

## Introduction

*Sanghuangporus* is an edible and medicinal fungal genus that has been used in China for centuries. The main biological components of *Sanghuangporus* include polysaccharides, flavonoids, and phenols. Research on this rare edible and medicinal fungus has revealed that some components in this mushroom have therapeutic effects against inflammation, tumors, and diabetes (1). Among them, polysaccharides exhibit strong immunomodulation and anticancer activity (2). Artificial cultivation has been successful in some species of *Sanghuangporus*, among them, *Sanghuangporus vaninii* (*S. vaninii*) is the most artificially cultivated variety. At present, artificial cultivation of *S. vaninii* is mainly carried out in greenhouses. With advancements in cultivation techniques, the cultivation of *S. vaninii* in a forest environment has been be successful. Cultivation of *S. vaninii* under a forest would not only avoid the cost of artificial greenhouses, but would also make a complete use of the natural shade conditions and the differences in day and night temperatures in a forest, thereby imitating the wild cultivation of *S. vaninii* and improving its quality.

Colorectal cancer is a common cancer disease with serious harmfulness, which is caused by an abnormal development of epithelial cells in the rectum and colon. Conventional treatments for colorectal cancer, such as surgery and chemotherapy, often lead to chemoresistance and side effects. Therefore, it is essential to discover new plant-derived therapies for colorectal cancer. Increasing attention is being paid to natural active substances as functional food and adjuvant therapies against colorectal cancer. Natural extracts have been proven to mediate the apoptosis of HT-29 cells in the caspase-3 pathway by reducing the expression of mutant p53 (3). It is believed that the antitumor activity of a biological macromolecule is associated with its specific structural characteristics and chain conformation. Therefore, it is important to clarify the inner connection between the conformation and biological activities of biomolecules.

Recently, some polymers obtained from *S. vaninii* with biological activities are increasingly attracting people's attention (3). However, compared with those cultivating in greenhouses, the structure and formational features of polysaccharides extracted from the *S. vaninii* under a forest have not been reported. Besides, the molecular mechanism of polysaccharide from the cultivated *S. vaninii* induced colon cancer HT-29 cells apoptosis and immunomodulation remained unclear. Therefore, we isolated a novel biomacromolecules from the cultivated mushroom of *S. sanghuang* under the forest and investigated its chemical properties by a series of techniques including GC-MS, methylation, Nuclear Magnetic Resonance, SEC-MALLS and Atomic Force Microscope. Moreover, the immunostimulation of this biopolymer and its potential mechanism of inducing tumor cell apoptosis *in vitro* were also explored.

## Materials and methods

### Materials and chemicals

The cultivated mushroom of *S. vaninii* was collected from Baiyansi forest, Hangzhou, China. A series of monosaccharide standards including rhamnose (Rham), ribose (Rib), arabinose (Ara), fucose (Fuc), xylose (Xyl), mannose (Man), glucose (Glc), galactose (Gal) were selected from Sigma Chemical Co., Ltd. (St. Louis, MO, USA). Primary and secondary antibodies against Bcl-2, Cytochrome c, β-actin, caspase3/9 and Bax were provided by Abcam Company (Cambridge, UK). DEAE-Sepharose Fast Flow and Sephacryl S-100 were selected from GE Healthcare (Chicago, IL, USA).

### Preparation of SVPS

The cultivated fruiting bodies of *S. vaninii* under forest were extracted with distilled water at 90°C for 2 h basing on the material-liquid ratio of 1:40 (w/v). The supernatants were obtained by centrifugation and then precipitated with five volumes of ethanol (95%). The Sevag reagent (n-butanol:chloroform, 1:4 v/v) was used to remove the protein from the extract. Ultimately, the supernatant was prepared into a crude polysaccharide (designated “SVPS”) using freeze-drying.

SVPS was isolated using chromatographic columns with different Separation characteristics In brief, the soluble polysaccharide (15 mg/mL) were loaded to the ion-exchange chromatography of DEAE Sepharose FF (6.0 × 50 cm) and eluted with a gradient of NaCl solutions (0–0.9 mol/L). The flow rate was 3.0 mL/min. The sugar content of each fraction was determined by phenol-sulfuric acid method (4). The collection was dialyzed, redissolved and transferred to a Sephacryl S-100 gel column (1.4 × 80 cm). After extensively dialyzing (above 7,000 kDa) and lyophilizing, the main polysaccharide component was collected (named SVPS2). The uronic acid in collections were detected by carbazole-sulfuric assay (5). Protein content was analyzed using Bradford method (6).

### Structuration and conformation of SVPS2

#### Physicochemical analysis

The chemical constituents of SVPS2 were identified by GC-MS technique according to the procedure presented earlier (7). Briefly, 2M trifluoroacetic acid (TFA) was used to degrade SVPS2 at 115°C for 2.5 h. NaBH_4_ was used to reduce the standard monosaccharides and hydrolysis sample. Then the mixture was neutralized by glacial acetic acid, and esterified with acetic anhydride at the same temperature for an hour. The well-prepared GC samples were analyzed after thorough trichloromethane-based extraction of the resulting mixture.

All the samples were injected on Agilent 7890B equipped with 5975C MSD system and a TG-5MS column (Agilent, 30 m × 0.25 × 0.5 mm). The analyzed conditions were set as following: the initial temperature was 120°C and increased up to 240°C at the rate of 3°C/min. The target temperature was 240°C for lasting 6 min.

#### FT-IR spectrum analysis

2 mg of SVPS2 was ground with dried KBr powder and transfered into a tablet for FT-IR spectroscopy. Its functional groups were recorded on a Fourier transformed infrared spectrometer (Nexus IS10 FT-IR; Thermo Nicolet, USA) with 64 scanning times and 4 cm^−1^ resolution in the frequency of 4,000–400 cm^−1^.

#### Analysis of methylation

Linkage pattern of SVPS2 was estimated by GC-MS as depicted previously described (8). In brief, polysaccharide (5 mg) was dissolved in a nitrogen-protected system containing NaOH-DMSO. The organic layers will appear in the obtained solution, and they will be extracted by dichloromethanefor three times.

Subsequently, the fraction was hydrolysed first with formic acid (90%), followed by hydrolyzed with 2 M TFA at 100°C for 3 h. After reducing by NaBH_4_, the dried hydrolyzed samples were acetylated with acetic anhydride at the same environment. Ultimately, the reacted products were converted into methylated glycol acetate (PMAAs) and analyzed by gas chromatography-mass spectrometry coupled with the same column described above. The temperature program was set at 150°C for 3 min, then increased to 250°C at 3°C/min, and kept at 250°C for 5 min. The injection port was set at 250°C.

#### NMR spectroscopy

After drying completely in a vacuum freezing dryer, 50 mg sample was exchange with D_2_O (0.6 mL) and put into an NMR tube before NMR analysis. The ^1^H, ^13^C and 2D homo- and heteronuclear NMR spectra were identified by a Bruker AVANCE spectrometer at 600 MHz. All the signal were recorded and analyzed with MestReNova 8.3 (9).

#### Advanced conformation

Some typical conformation parameters such as number-average (*M*n) and molecular weight (*M*w) were analyzed using a size exclusion chromatography along with a multi-angle laser light scattering system (SEC-MALLS, Wyatt Technology, USA). The intrinsic viscosity [η] was acquired by an online differential viscometer (ViscoStar^TM^ II, Wyatt Technology, USA). The SEC column [TSK-gel 3000PWXL column (7.8 × 300 mm)] was used. After filtering by 0.22 μm membrane, 1 mL sample (2 mg/mL) was put into the chromatographic system equipped with a SEC column [TSK-gel 3000PWXL column (7.8 × 300 mm)]. A mobile phase containing 0.1 M NaNO3 and 0.02% (w/w) NaN_3_ was used at the flow rate of 0.5 mL/min. All the data was analyzed by ASTRA software and the parameter (*dn/dc*) was 0.138 mL/g (2).

AFM was used to visualize microstructures of sample following the previous reference (10). In brief, a stock solution of SVPS2 was prepared with ultrapure water, and then gradually diluted to a final concentration of 15 μg/mL. SVPS2 solution (5 μL) was transferred onto a freshly cleaved mica. Si_3_N_4_ probe and 0.21 N/S elastic modulus were adopted. Ultimately, the microcosmic images of SVPS2 were scanned and acquired by AFM (XE-70), using commercia cantilever in tapping mode.

#### Immunomodulation of SVPS2

RAW 264.7 cells were cultivated in a high-sugar DMEM media containing 10% FBS and 1% penicillin-streptomycin, which were grown in a 5% CO_2_ atmosphere at 37°C. The cell viability of each treated macrophages was determined by CCK-8 assay (11).

The RAW 264.7 cells were pre-incubated at a density of 5 × 10^4^ cells/mL per well. Control cells were not treated with samples and LPS. Various concentrations of polysaccharides (50–600 μg/mL) and LPS (1 μg/mL) as the positive control were transferred into each well, and incubated for 24 h at 37°C. The secretion of NO was performed by Griess method. The released cytokines of TNF-α, IL-1β, and IL-6 in each group were tested by ELISA kits. All treatments were determined in triplicate and the data were expressed as mean ± SD.

### Antitumor assay

#### Cell viability

Colorectal cancer cells (HT-29), obtained from Procell Life Science aaand Technology Co., Ltd., (Wuhan, China) were cultured in the same conditions as RAW264.7 cells. The cell viability of HT-29 cells treated with SVPS2 was evaluated by MTT assay (12). Briefly, cells were digested in trypsin and transferred into the culture medium. Subsequently, the cells were added to a 96-well plate and the density was controlled at 5 × 10^4^ cells/ml. After 24 h, the cells were incubated with SVPS2 ranging from 0 to 600 μg/mL using 5-Fu (5-fluorouracil) as positive control. Cells in the negative control group were cultured only with RPMI-1640 medium. MTT (20 μL) was injected into the wells, in which cells were incubated for 4 h. After taking off the supernatant, an aliquot of 200 μL DMSO was injected to each well. The microplate reader was used to record the absorbance at 570 nm.

#### Detection of apoptosis by flow cytometry

The Annexin V-FITC/PI double staining method was used to analyze the apoptosis of HT-29 cells treated with polysaccharides (13). In brief, HT-29 cells (2 × 10^5^ cells/well) were inoculated in each plate and incubated in a constant temperature incubator at 37°C. Then they were exposed to different doses of SVPS2 for 24 h. The treated cells were collected followed by washing thrice with cold PBS and subjected to be labeled with Annexin V-FITC and PI in the dark. Ultimately, the resulting samples were determined using a flow cytometer.

#### Determination of reactive oxygen species (ROS)

The Dichloro-dihydro-fluorescein diacetate (DCFH-DA) assay was used to detect the level of intracellular ROS generation (14). Briefly, HT-29 cells were plated at a density of 1 × 10^6^/ mL in 12-well plates and incubated at 37°C for 24 h. After that, different concentration of SVPS2 (0–600 μg/mL) were stained with 10 μmol/L DCFH-DA for 30 min at 37°C in darkness. Finally, cells were washed thrice with PBS to remove any residual DCFH-DA. The fluorescence intensity was determined using a fluorescence microscope (ex/em, 488/525 nm).

### Cell cycle distribution assay

The PI stained-cell cycle was carried out following the reported reference with minor modifications (15). Briefly, HT-29 cells were added to in 12-well plates and exposed to different concentration of SVPS2 in the range of 0–600 μg/mL. When the cells were treated for 24 h, they were transferred and maintained in iced fixing buffer (70% ethanol in PBS). After another washing thrice, the cells were replaced with PBS having 50 μg/mL of ribonuclease (RNAse) and cultured at 37°C for 0.5 h. After this procedure, the cells were then stored on ice and 400 μl PI was added to each sample. Finally, the proportions of stained cells were determined and processed by ModFit LT software.

### Western-blot examination

It was carried out according to the method published earlier (15). HT-29 cells were exposed to different doses of SVPS2 (0–600 μg/mL) and total protein was extracted using cell lysis buffer containing 1% PMSF, protease and phosphatase inhibitors. Protein content was analyzed using a bicinchoninic acid (BCA) kits. Thirty micrograms of proteins were fractionated by 12% SDS-PAGE followed by transferred to 0.45 μm PVDF membranes. After blocking for 4 h with 5% non-fat milk, the membranes were incubated with primary antibodies (Bax, Bcl-2, cleaved caspase 3, cleaved caspase 9) in TBST (dilution at 1:1,000) overnight at 4°C. Following three washes with the TBST, the membranes were incubated with the corresponding secondary antibodies (goat anti-rabbit IgG H&L) at a dilution of 1:5,000 for 1 h. After another washing, the protein bands were visualized using a chemiluminescence reagent (ECL) reagents. The Image Lab software was used to measure the protein expression levels.

### Statistical analysis

All experiments were performed at least thrice and the data were expressed as mean ±SD. The statistical significance was calculated by one-way variance analysis (ANOVA). The *p*-value <0.05 was regarded as statistically significant.

## Results and discussion

### Preparation, purification and chemical compositions

**Figure 1:**
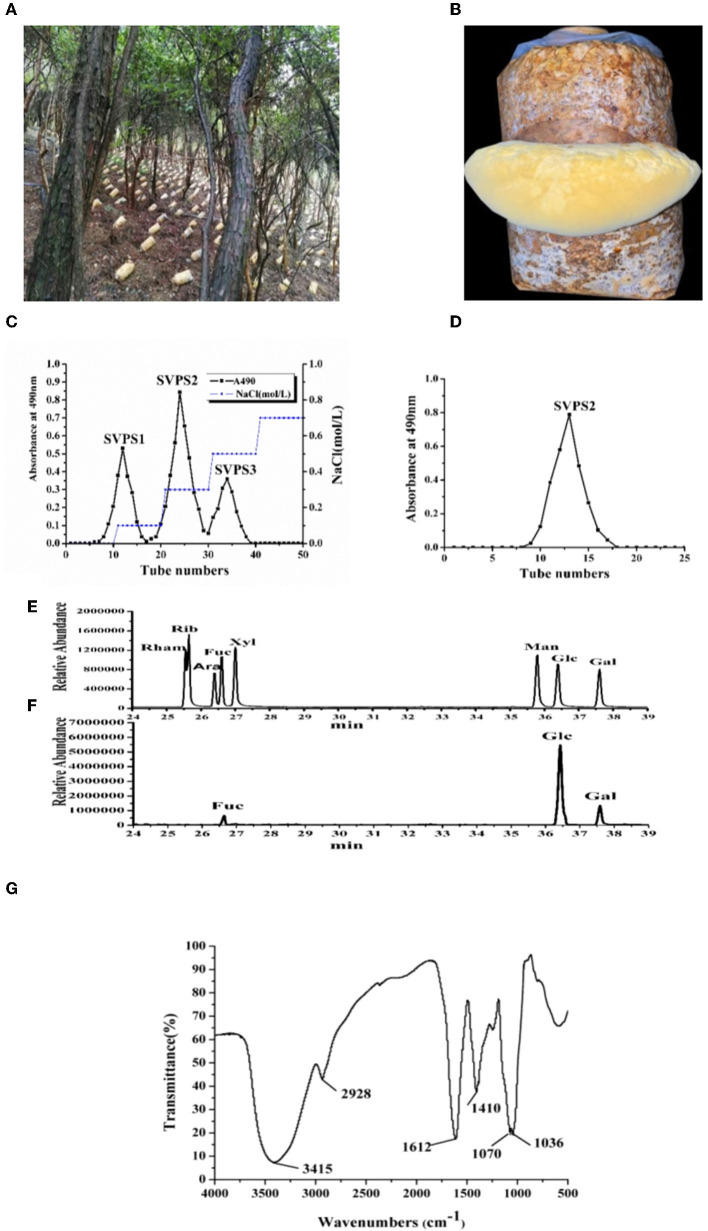
The on-site picture of cultivated fruiting bodies **(A)** and the enlarged part of S. vaninii in forest **(B)**. The profile of crude polysaccharides eluted on DEAE-Sepharose Fast Flow column **(C)** and the purified SVPS2 fraction by Sephacryl-S 100 column **(D)**. The GC-MS results of standard sugars **(E)** and PMP-derived SVPS2 **(F)**. IR spectrum **(G)**.

The fruiting bodies of *S. vaninii* were collected from cultivation under the forest (Figures 1A, B). The yield of crude polysaccharide (SVPS) obtained by extraction and deproteinization was 1.52%. After DEAE-Sepharose FF column chromatography, three fractions were collected and designated as SVPS1 (27.7%), SVPS2 (51.2%), and SVPS3 (21.1%), respectively (Figure 1C).

Antioxidant experiments including the scavenging capacity of superoxide radical and ABTS+ free radical demonstrated that the activity of SVPS2 was the highest among the three components. Consequently, we mainly explored the activities and conformation of SVPS2. Finally, SVPS2 (Figure 1D) was further purified by Sephacryl S-100 gel column. SVPS2 contained 0.72% protein and no uronic acid was tested.

The monosaccharide composition analysis was applied to understand the structural features. As shown in Figures 1E, F, SVPS2 was a neutral heteropolysaccharide which was mainly composed of glucose, galactose and fucose in a ratio of 13.9:2.7:1.0. There was obvious difference between the monosaccharide composition of SVPS2 and other polymers obtained from *Sanghuangporus* (3). Water-soluble polysaccharide (SVP) from *S. vaninii* cultivated in greenhouse consisted of mannose, rhamnose, glucuronic acid, galacturonic acid, glucosamine, glucose, galactosamine, galactose, xylose, arabinose, and fucose in molar ratios of 1.63:0.04:0.36:0.03:0.13:8.39:0.08:1.08:0.25:1.07:0.40. Various adverse environmental stresses, such as UV, low temperature and temperature difference change can induce the synthesis of secondary metabolites in plants and fungi (16, 17). Therefore, the particular monosaccharide composition of SVPS2 ascribes to the specific strains and the induction of cultivation environment under the forest.

It was obvious that the IR spectrum of SVPS2 showed not only the basic functional groups of polysaccharides but the intrinsic characteristics of furanose rings in Figure 1G. Among them, the presence of near 3,410 cm^−1^ was attributed to the O–H stretching vibration band and the absorption peak at 2,930 cm^−1^ was due to the C–H stretching vibration (18). The strong peak at around 1,606 cm^−1^ was attributed to the C=O stretching vibration, while the signal at approximately 1,401 cm^−1^ was ascribed to C-H bending vibration. Attractively, the appearance of only two absorption peaks at 1,070 and 1,038 cm^−1^ in the range of 1,000–1200 cm^−1^ confirmed the existence of furanose units in SVPS2 (19, 20). Those results were in accordance with the analysis of monosaccharides.

### Structural property

#### Glycosidic analysis

The linkage status of glycosyl residues of SVPS2 was determined by methylation analysis, which was listed in Table 1. The results indicated that it was mainly composed of 1,5-linked Glc*f* (44.62 mol%), 1,5,6-linked Glc*f* (27.96 mol%), terminal Glc*f* (5.93 mol%), terminal Gal*p* (17.21 mol%) and terminal Fuc*p* (4.28 mol%), which was consistent with its monosaccharide. The glycosidic linkages of SVPS2 was different from those of the polysaccharides from *Sanghuangporus vaninii* cultivated in greenhouses, such as SVP-1, whose main chain contains → 3)-Glc*p* (1 →, → 6)-Glc*p* (1 →, → 4)-Glc*p* (1 →, → 6)-Gal*p* (1 → and → 3, 6)-Glc*p* (1 → (3).

**Table 1:** Linkage patterns of SVPS2 by methylation and GC-MS.

**Methylated sugars**	**Linkages patterns**	**Molar ratio (%)**	**Major mass fragments (*m/z*)**
2,3,4,6-Me_4_-L-Fuc*p*	1-linked Fuc*p*	4.28	43,59,71,89,101,117,129,144,161, 187
2,3,5,6-Me_4_-D-Glc*f*	1-linked Glc*f*	5.93	43,59,87,89,101,113,117,205
2,3,4,6-Me_4_-D-Gal*p*	1-linked Gal*p*	17.21	43,87,99,102,117,127,205
2,3,6-Me_3_-D-Glc*f*	1,5-linked Glc*f*	44.62	43,87,99,101,117,127
2,3-Me_2_-D-Glc*f*	1,5,6-linked Glc*f*	27.96	43,87,101,117,127

#### NMR analysis

**Figure 2:**
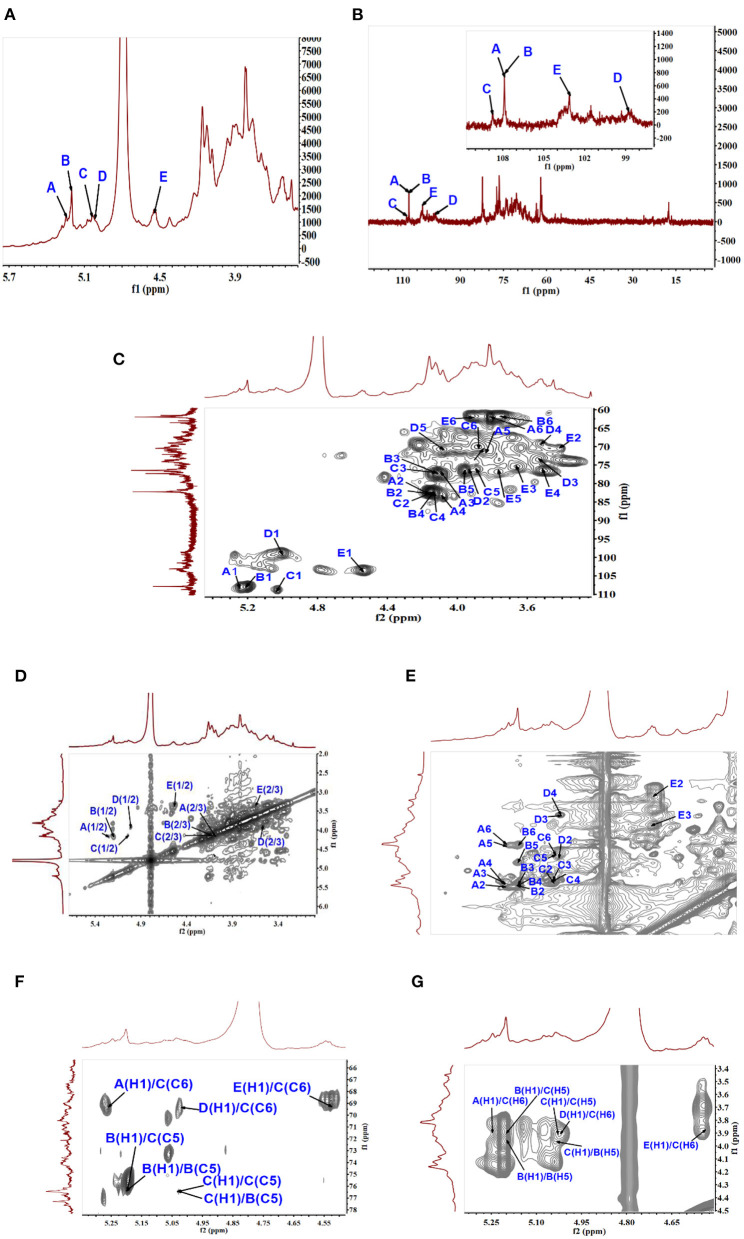
NMR analysis of SVPS2 structure. **(A)** ^1^H NMR spectroscopy, **(B)** ^13^C spectrum, **(C)** HSQC spectrum, **(D)** ^1^H-^1^H COZY spectrum, **(E)** TOCSY spectrum, **(F)** HMBC spectrum, **(G)** NOESY spectrum in the anomeric region.

1D and 2D NMR spectra were employed to further identify the correlation between glycosidic residues of SVPS2. Five strong cross peaks at δ 5.25/107.88, δ 5.20/107.88, δ 5.03/108.80, δ 5.00/98.74, and δ 4.53/104.11 ppm were found in HSQC spectrum, which were designated as residues A–E, respectively (Figures 2A–C). The existing of carbon signals at δ 82.00–84.00 ppm demonstrated that there were furanosidic residuces in SVPS2 (21). Among them, the dominant anomeric signals were signed at 107.88 ppm and 108.80 ppm, suggesting the presence of β-glucofuranose (22). The major chemical shifts, linkage analysis, and the proton and carbon chemical shifts are summarized in Table 2.

**Table 2:** Chemical shift assignments of 1D and 2D NMR of SVPS2.

**Residues**	**Chemical shifts**, δ **(ppm)**					
	**H-1/C-1**	**H-2/C-2**	**H-3/C-3**	**H-4/C-4**	**H-5/C-5**	**H6a, H6b/C6**
(A) β-Glc*f*-(1 →	5.25/107.88	4.16/82.15	4.08/77.34	4.08/83.59	3.82/71.20	3.67,3.81/63.47
(B) → 5)-β-Glc*f*-(1 →	5.20/107.88	4.16/82.15	4.12/77.34	4.14/82.13	**3.96/76.39**	3.74,3.81/61.98
(C) → 5,6)-β-Glc*f*-(1 →	5.03/108.80	4.14/82.15	4.12/77.34	4.12/82.45	**3.89/76.26**	**3.89,4.22/69.55**
(D) α-Fuc*p*-(1 →	5.00/98.74	3.92/76.72	3.54/73.78	3.52/69.30	4.08/70.80	1.25/17.58
(E) β-Gal*p*-(1 →	4.53/103.11	3.42/70.82	3.67/75.53	3.52/76.90	3.78/76.91	3.91/62.02

The bold values indicated to emphasize the downfield shift of C-5 and C-6.

In residue A, the anomeric proton at δ 5.25 ppm had close relationship with the carbon signal at δ 107.88 ppm. According to the reported values (23), no ^13^C shifts were induced by glycosylation. Therefore, it was endorsed as β-D-Glc*f*-(1 →. Residual B showed strong anomeric signals at 5.20 ppm and 107.88 ppm, suggesting that it was Glc*f* unit and had a relatively high content (24). All the chemical shifts of H-1 to H-6 and C-1 to C-6 were obtained from the COZY (Figure 2D) combined with TOCSY (Figure 2E) and HSQC (Figure 2C). Based on the chemical shift of reference analogous compounds, the downfield shift of C-5 (δ 76.39 ppm) led to the identification of residue B as → 5)-β-D-Glc*f*-(1 →. The obvious downfield carbon resonances at C5 (δ 76.26 ppm) and C6 (δ 69.55 ppm) indicated that Residue C, with H-1/C-1 signals at δ 5.03/108.80 ppm, was pointed to be → 5, 6)-β-D-Glc*f*-(1 → (24). The anomeric chemical shifts at δ 5.00 and δ 98.74 ppm illustrated that Residue D was α-anomer. The assignment of the residue D as α-L-Fuc*p*-(1 → was done by two characteristic peaks of δ 1.25 and δ 17.58 ppm (25), which belong to the CH3–C group resonance signals. Residue E was deduced to be β-D-Gal*p* from the cross peaks at δ 4.53/103.11 ppm, which agreed with literature data (26).

The intra-connections between neighboring glycosyl residues of SVPS2 was illustrated from the HMBC (Figure 2F) combined with NOESY (Figure 2G). The strong connectivity in HMBC was found from B H-1/B C-5 (5.20 /76.39 ppm) spectrum, implying that the → 5)-β-Glc*f*-(1 → residue was linked to → 5)-β-Glc*f*-(1 →. The connection between C H-1 and C C-5 in the HMBC implied that → 5, 6)-β-Glc*f*-(1 → residue was connected to → 5, 6)-β-Glc*f*-(1 →. The inter-cross linkages were observed from B H-1/C C-5 (δ5.20/76.26 ppm), indicating that → 5)-β-Glc*f*-(1 → residue was connected to → 5, 6)-β-Glc*f-*(1 →. Simultaneously, the cross-peaks was identified from C H-1/B C-5, suggesting that → 5, 6)-β-Glc*f*-(1 → residue was linked to → 5)-β-Glc*f*-(1 →.

The connectivity was detected from E H-1/C C-6 (4.53 /69.55 ppm) in the HMBC spectrum, implying that residue E was linked to the C-6 position of residue C on the backbone. The H-1 signal of A was correlated to the C-6 signal of C, suggesting that residue A was linked to the C-6 position of residue C. Similarly, the HMBC connects D H-1 to C C-6, implying the sequence D-(1 → 6)-C. The above sequences were further verified by NOESY spectrum.

**Figure 3:**
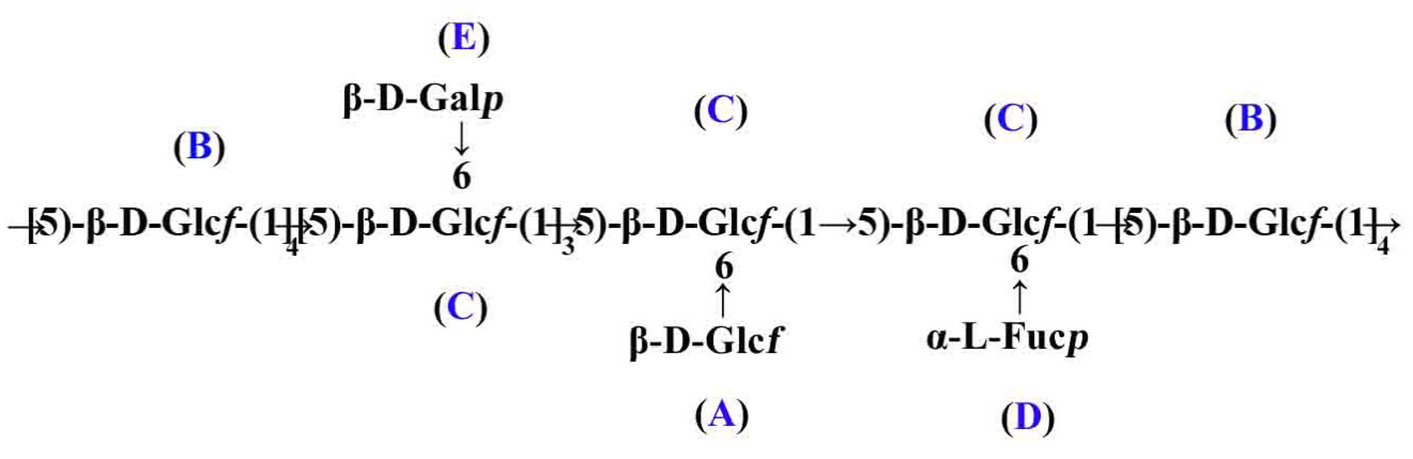
Presumed main structure of SVPS2.

Taken together, the putative primary structure of SVPS2 was established as shown in Figure 3. It was a heterogeneous group of β-glucofuranose polymers with → 5)-β-Glc*f*-(1 → as backbone and branches at C-6 by terminal β-D-Gal*p*, β-D-Glc*f*, and α-L-Fuc*p* residues, which was extremely distinct from other macromolecules isolated from different resources of *Sanghuangporus*.

We previously reported a 1, 6-linked α-D mannogalactan, which was isolated from the fruiting body of *Sanghuangporus sanghuang* growth in a wild environment (27). Another polysaccharide (SVP-1), which was extracted from the main parts of *Sanghuangporus vaninii* cultivated in greenhouses, contained a 1, 4 and 1, 6-linked β-D-Glc*p* residues backbone (3). After man-made liquid cultivation, a 1, 4-linked α-D-glucan was obtained from the mycelia of *Sanghuangporus sanghuang* (28). Meanwhile, a 1, 3-linked and 1, 2-linked α-D-mannan appeared in the culture broth (2).

Intriguingly, a series of different sugar rings were detected in the reclaimed biomacromolecules from the various parts of *Sanghuangporus* prepared in diverse cultivation modes. Polysaccharide obtained from the mycelia of *Sanghuangporus* by fermentation had a tendency to form α-configuration glucose, while the polysaccharide from the fruiting body of *Sanghuangporus* tends to form the β-configuration The results helped to draw a conclusion that the cultivation under forest may attribute to the formation of β-configuration and furan ring of glucose compared with the liquid fermentation and cultivation in greenhouses. The reason might be explained that the different environment including light intensity, moisture and temperature could be beneficial for the biosysthesis of β-glucofuranose by controlling some critical enzymes (16).

### Chain conformation of SVPS2

**Figure 4:**
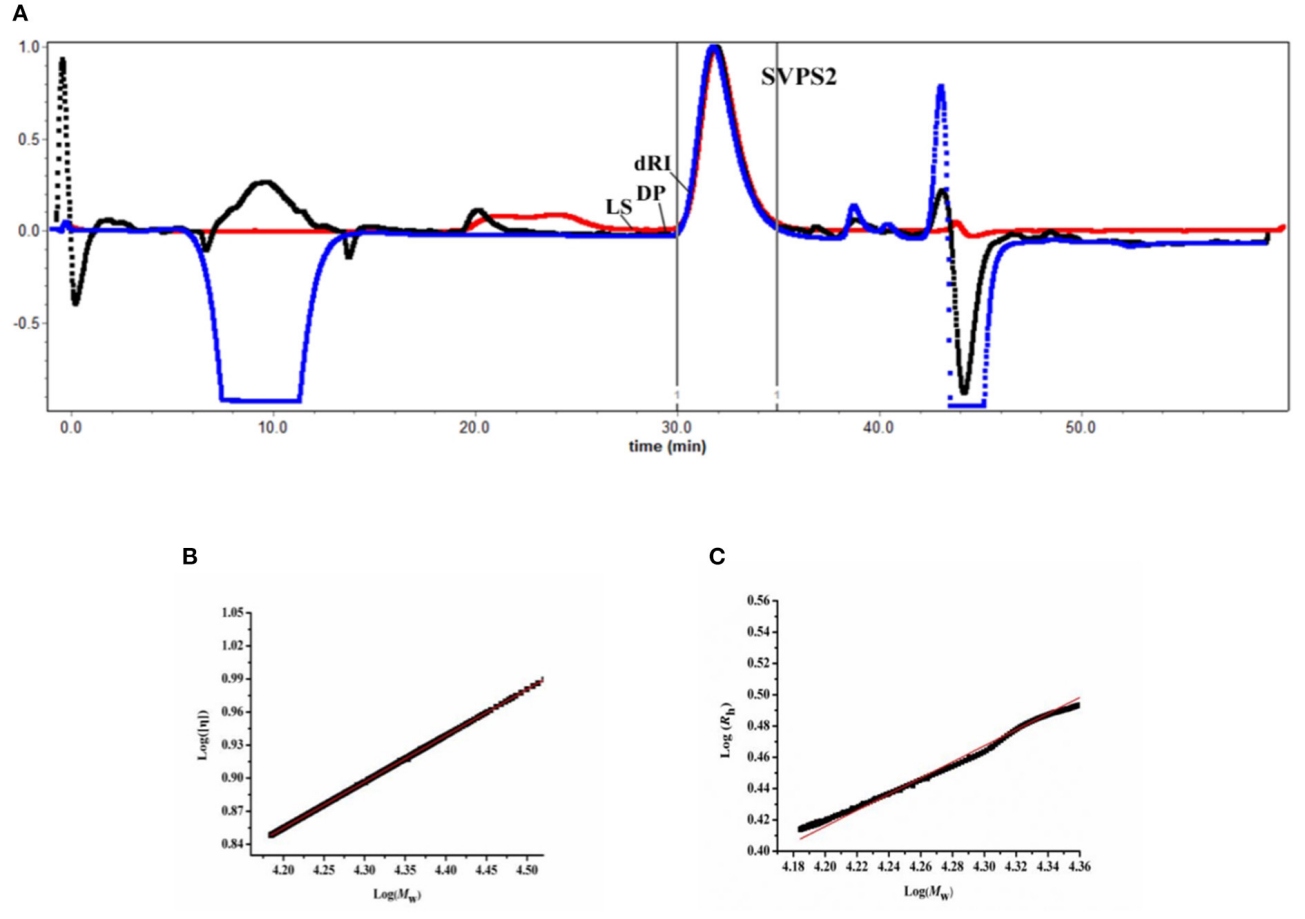
Size exclusion chromatography profile of SVPS2 **(A)**, Double logarithmic plot of intrinsic viscosity (η) vs. molecular weight (*M*w) **(B)** and hydrodynamic radius vs. molecular weight **(C)** for SVPS2.

To analyze the conformation of polymer in solution, the acromolecular parameters of SVPS2 were characterized by SEC-MALLS-Vis system. According to SEC chromatographic analysis, only a single symmetrical peak appeared in the graph, demonstrating that SVPS2 was a homogeneous polymer (Figure 4A). The absolute *M*w of SVPS2 was detected to be 23.4 kDa with 3.17 nm of *R*h. SVPS2 displayed a narrow molecular weight distribution based on the polydispersity index of it was 1.07.

The classical Mark-Houwink equation ([η] = *KM*_w_^α^) was employed to determine the intrinsic viscosity [η] of SVPS2 in solution by analyzing the value of α. The Mark-Houwink exponent a value was measured to be 0.537 using plotting log [η] vs log *M*w (Figure 4B), indicating that SVPS2 exhibits as a flexible chain conformation in saline solution. Moreover, the exponent v value of 0.521 was calculated basing on the plot of *R*h vs. *M*w (*R*_h_= $KM_{\text{w}}^{\text{v}}$) (Figure 4C), which further confirmed the existence of a flexible chain (29).

### Analysis of microstructure by AFM

**Figure 5:**
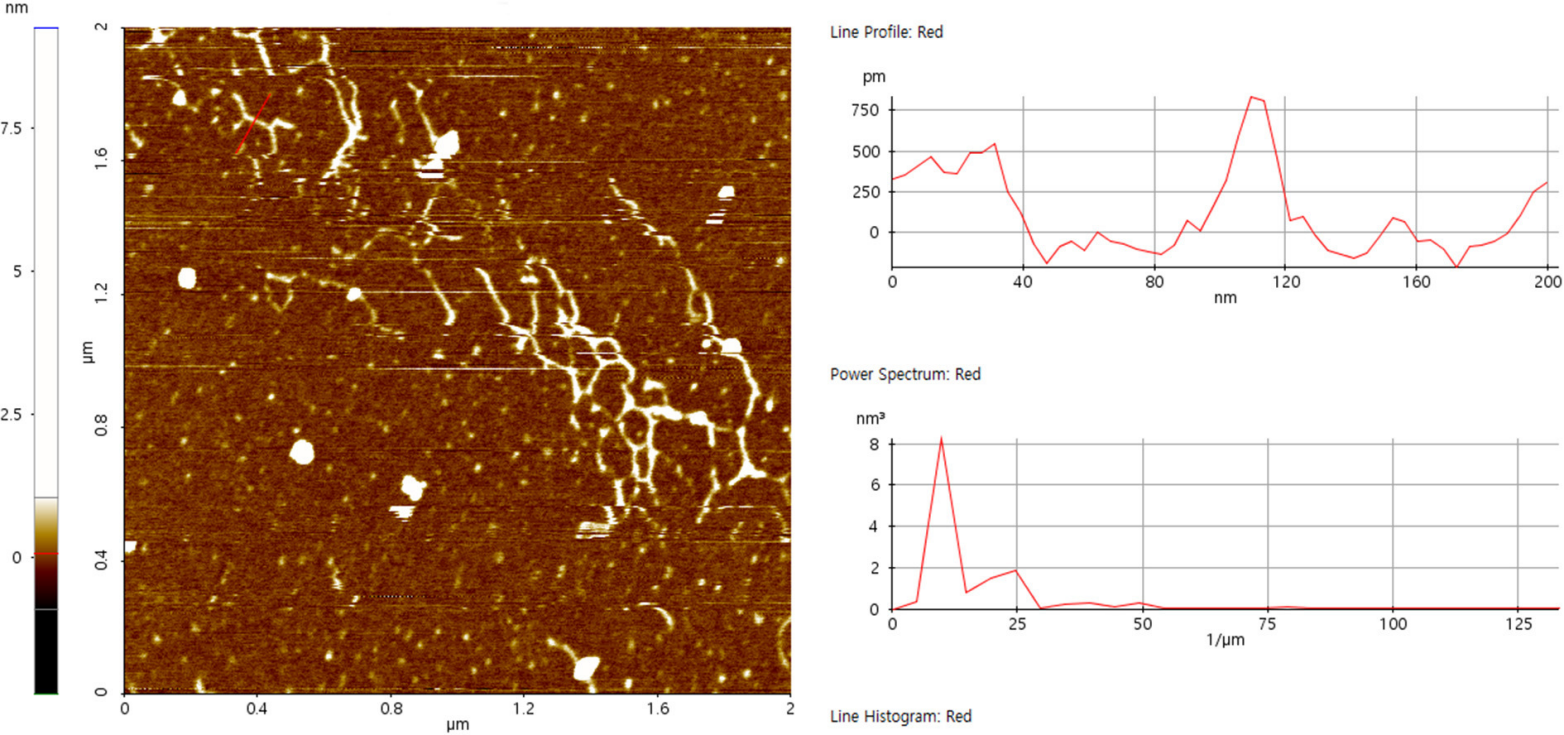
Atomic force micrographs of SVPS2.

High precision morphology can be acquired at nanometer scale by AFM, which provides direct evidence for molecular conformation of biopolymers. After diluting to 30 μg/mL, some of the polysaccharide chains were entangled and accompanied by multiple branches, forming a certain degree of accumulation (Figure 5). The single-stranded height of the chain was 0.72 ± 0.08 nm, implying that SVPS2 exhibited an extended flexible conformation (30).

### Immunoregulatory activity and antitumor of SVPS2

#### Immunomodulatory activities of SVPS2 on RAW 264.7 cells

Macrophages are involved in the innate immune response, which can participate in host defense against pathogens and the invasion of cancer cells. The antitumor activities of biomacromolecule is closely associated with regulation by immune intervention (31). For investigation of the viability of RAW 264.7 macrophages, it was hard to find any comparison between sample groups and the control (*p* > 0.05), which indicated that SVPS2 had no toxicity against RAW 264.7 cells *in vitro*.

**Figure 6:**
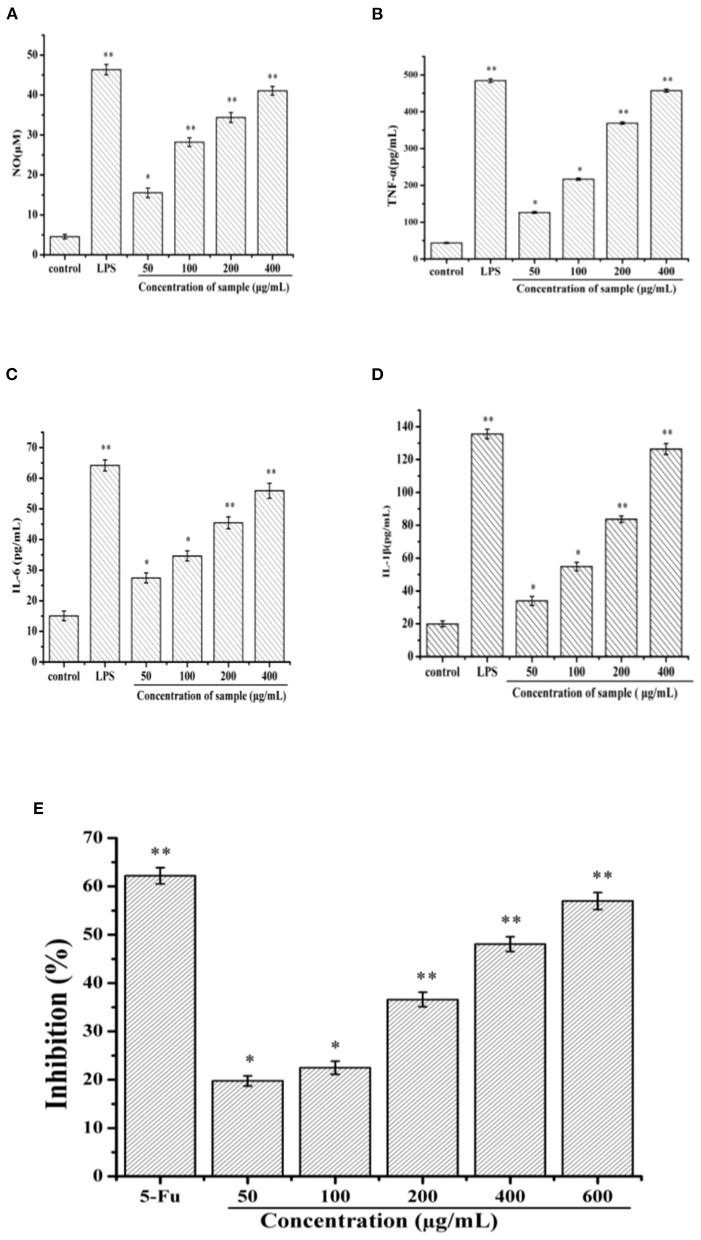
Effects of SVPS2 on the **(A)** NO, **(B)** TNF-α, **(C)** IL-6, and **(D)** IL-1β secretion of RAW264.7 macrophages. **(E)** Cell viability of HT-29 treated with SVPS2. *p < 0.05, **p < 0.01 compared to the control group.

NO is an important signal transduction medium in immune regulation and could induce the death of tumor cells (11). As shown in Figure 6A, SVPS2 could significantly improve the secretion of NO in a concentration-dependent manner, indicating that it might possess the ability to eliminate tumor cells. After intervention, the treated cells may recruit a variety of signals including TNF-α, IL-1β and IL-6 to help activating immune system, which would be beneficial for defense of tumor cells. Compared with that of control group, SVPS2 significantly promoted the secretion of TNF-α, IL-6 and IL-1β in a concentration-dependent manner ranging from 50 to 400 μg/mL (Figures 6B–D). The results indicated that SVPS2 could facilitate the initiation of the immune reaction and increased the production of cytokines in immune cells.

#### Effect of SVPS2 on the inhibition of HT-29 cells

Polysaccharides derived from plants and fungi significantly inhibited the growth of cancer cells (12). In this study, the viability of SVPS2 on HT-29 cell line was tested *in vitro* by MTT assay. Within the concentration range of 50–600 μg/mL, the inhibitory effect of SVPS2 on HT-29 cells increased in a dose-dependent manner (Figure 6E). The inhibition rate increased from 19.75 to 56.97%, indicating that SVPS2 exhibited obvious inhibitory effect on HT-29 cell proliferation. Interestingly, SVPS2 (600 μg/mL) markedly inhibited the proliferation of HT-29 cells, which was as close as the ability of 5-Fu (50 μg/mL). The IC_50_ of SVPS2 toward HT-29 cells was 483 μg/mL in 24 h. The results demonstrated that SVPS2 exhibited an antitumor activity in HT-29 cells with negligible toxicity on normal cells (Figure S1), and enhanced the immune function of RAW 264.7 cells. Considering the different cultivation environment of the cultivated fruiting body of *S. vaninii* under forest compared with those in greenhouse, the immunoregulatory and anticancer activities of SVPS2 may be associated with its unique monosaccharide composition and conformation. It has been proved that polysaccharides can provoke immune system response *via* enhancing the immunoregulation of host, exhibitng its antitumor activity. Cai et al. reported that a novel polysaccharide (SP90–1) purified from *Spirulina platensis* enhanced immunity on RAW264.7 cells and significantly inhibited the growth of A549 lung cancer cells (32). It indicated that SVPS2 significantly inhibited the growth of HT-29 colorectal cancer cells, which was associated with the promotion of immune regulation.

#### Effect of SVPS2 on ROS generation

**Figure 7:**
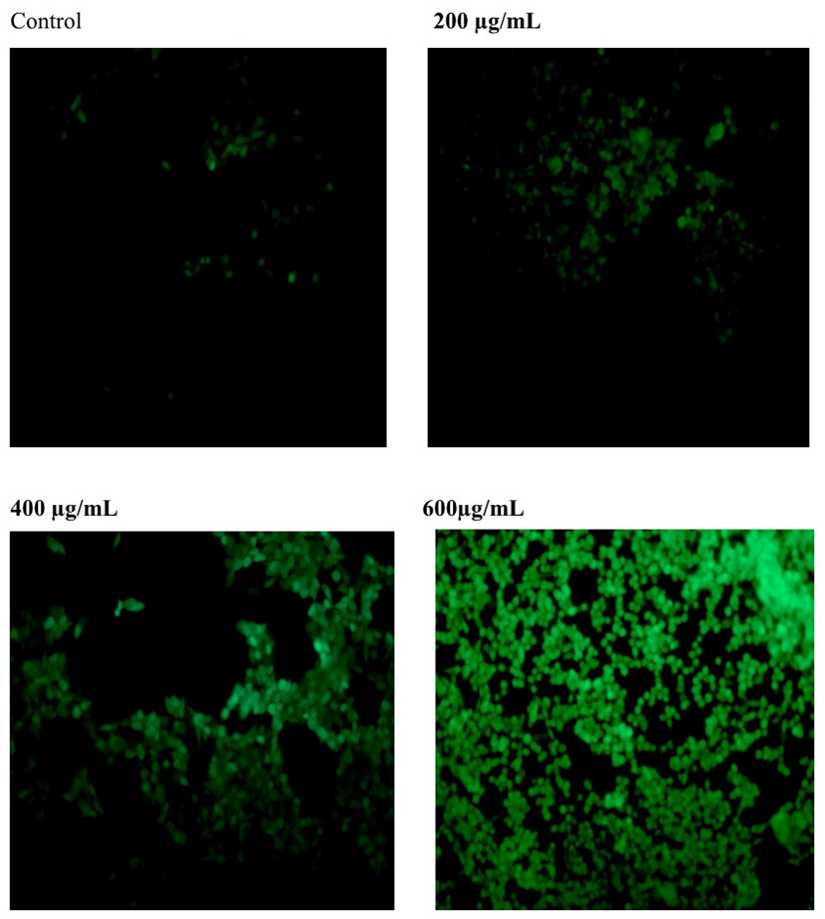
Effect of SVPS2 on ROS production in HT-29 cells.

Reactive Oxygen Species (ROS) can be regarded as the initiation factor of apoptosis, which can regulate the downstream apoptosis signaling pathway (14). As shown in Figure 7, the fluorescence intensity of the control group was relatively weak. After treatment with different concentration of SVPS2, the fluorescence intensity significantly increased compared with the control group. The results indicated that SVPS2 could stimulate ROS generation within HT-29 cells in a dose-dependent manner and further induced the cell apoptosis.

#### SVPS2-induced apoptosis in HT-29 cells

**Figure 8:**
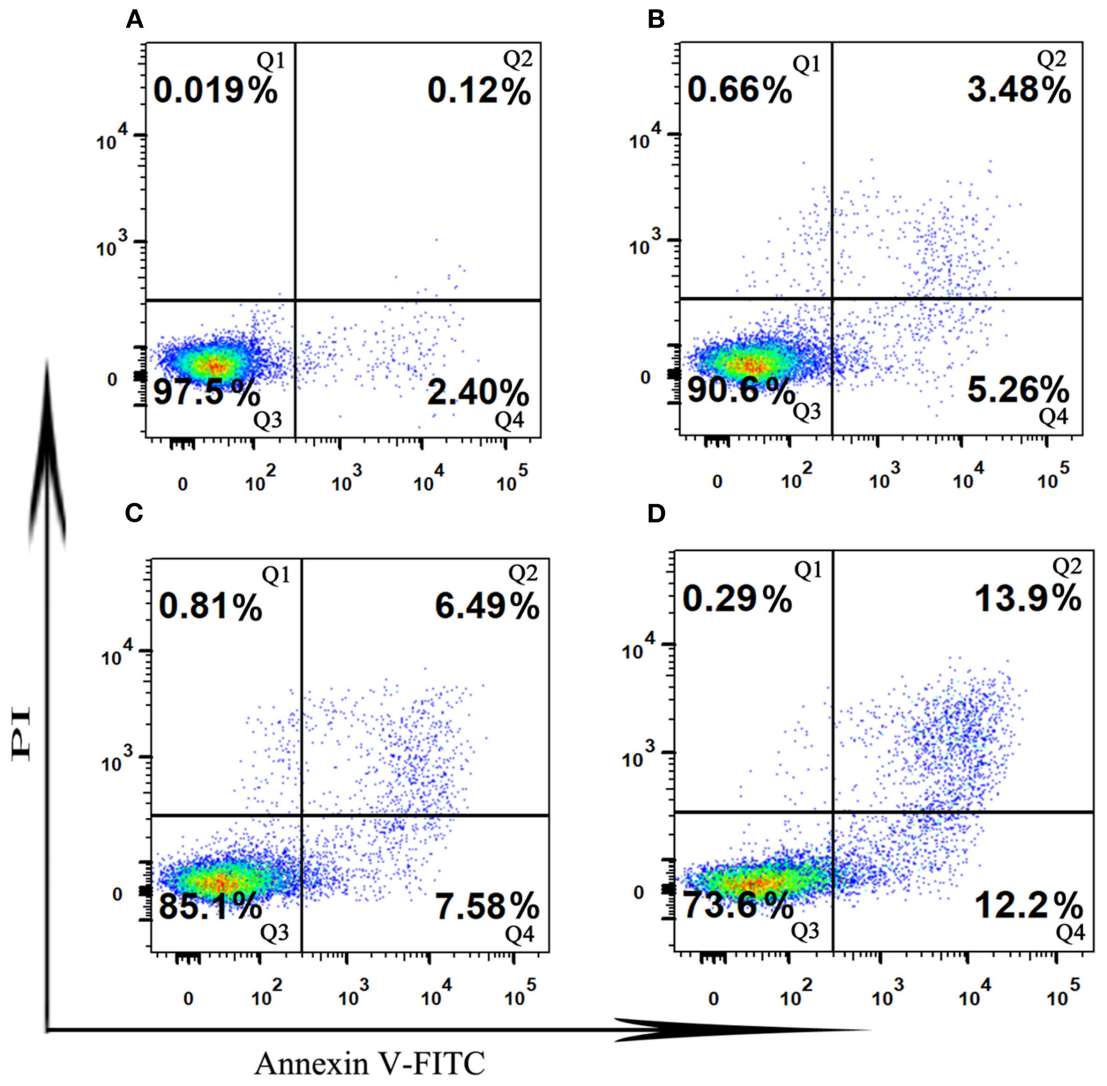
The cell apoptosis in HT-29 cells exposed to different concentration of SVPS2 **(A)** 0, **(B)** 200, **(C)** 400 and **(D)** 600 μg/mL after 24 h. Q1–Q4 quadrants represent: Q1, cells stained with PI (necrotic cells); Q2, cell conjugated with Annexin V and stained with PI (late apoptotic cells); Q3, unconjugated with Annexin V and unstained with PI cells (viable cells); Q4, cells conjugated with Annexin V (early apoptotic cells).

Induction of apoptosis is an important physiological and crucial process, which is considered to be an important mechanism of antitumor drugs can act. Herein, flow cytometry attached to V-FITC/PI was used to determine early and late apoptosis of HT-29 cells induced by SVPS2. As shown in Figure 8, the proportions of apoptotic cells exhibited a concentration-dependent increase in early (from 2.4% to 12.2%) and in late (from 0.12 to 13.9%) when the initial concentration of SVPS2 went up to 600 μg/mL. These data indicated that SVPS2 could induce apoptosis of HT-29 cells, which was in accordance with the results of MTT analysis.

### Cell cycle

**Figure 9:**
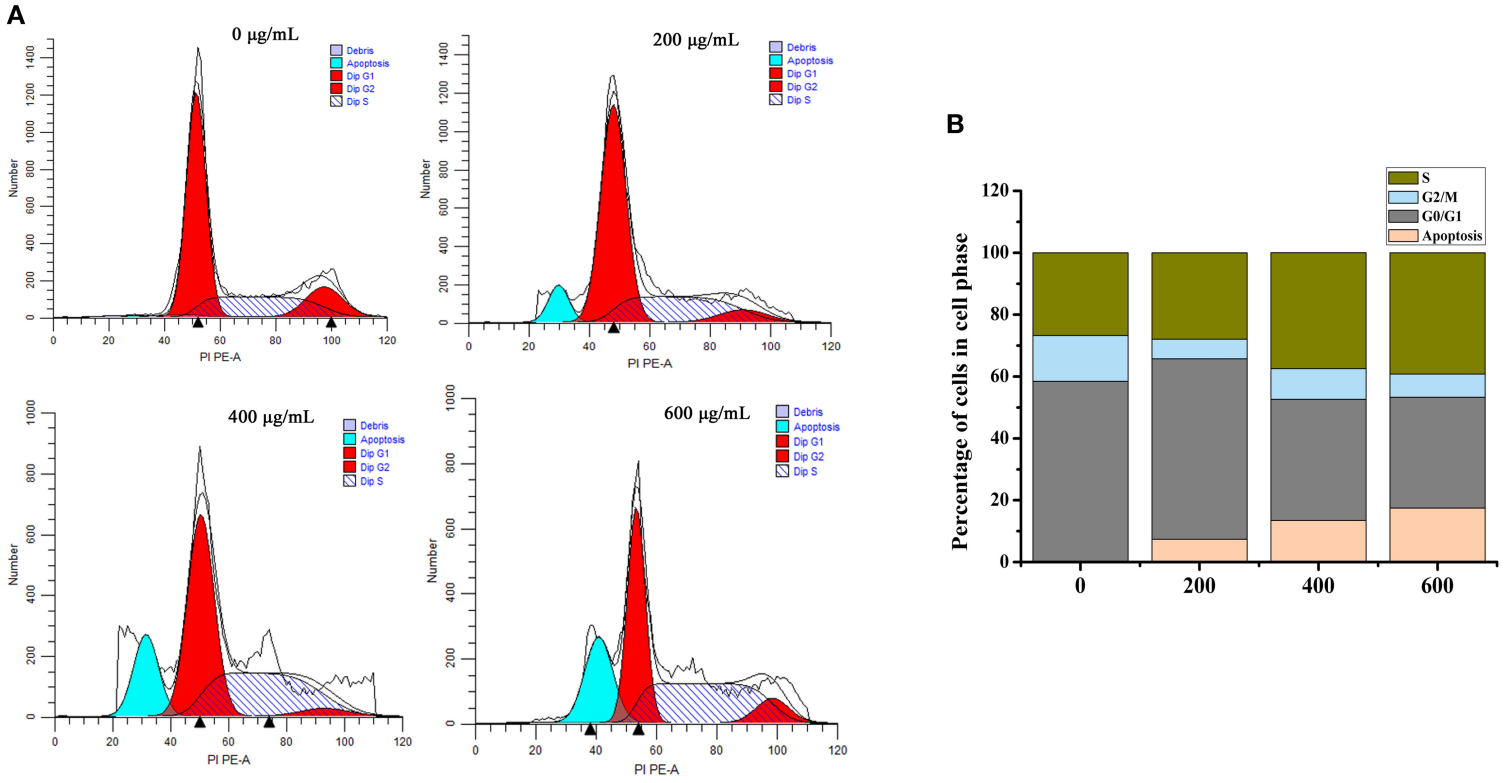
Effects of SVPS2 on cell cycle distribution of HT-29 cells. **(A)** The percentage of HT-29 cells in each cell cycle phase after treatment with SVPS2. **(B)** Proportion chart of each cycle.

The apoptotic cell cycle could be conducted by analyzing the periodic distribution of PI-stained HT-29 cells exposed to the sample. Compared to the untreated control, the S phase cell population increased to 39.21% after treatment with SVPS2, with an associated decrease in the G0/G1 phase population (Figure 9). These data illustrated that SVPS2 caused cell cycle arrest at the S phase. Similarly, a neutral polysaccharide (GFP-A) isolated from *Grifola frondosa* induced apoptosis in HT-29 cells and then induced cell cycle in the S phase (33). Other polysaccharides, such as SPS-CF, which was a sulfated glucuronorhamnoxylan polysaccharide derived from *Capsosiphon fulvescens*, induced cell death by arresting the HT-29 cells at G2/M phase.

### Western blot analysis

**Figure 10:**
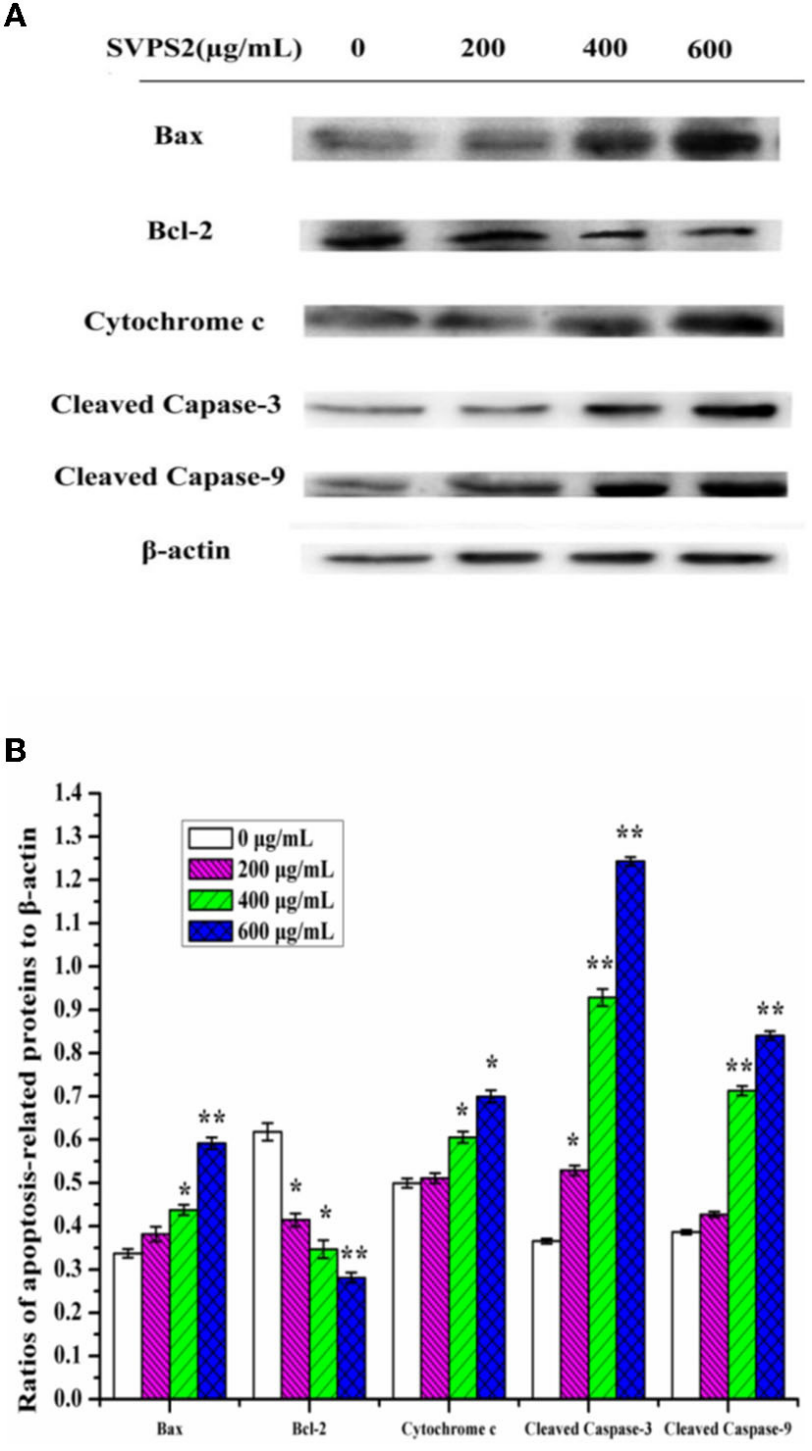
**(A)** Expressions of proteins of SVPS2-treated HT-29 cells. **(B)** Relative protein levels were expressed normalized to β-actin. The * symbol indicate the values of *p* < 0.05 and the ** symbol indicate the values of *p* < 0.01 compared to the control group.

Western blot was employed to understand the expression levels of apoptosis-related proteins. Bax and Bcl-2 are the two key regulators that control the release of related factors in mitochondrial-mediated apoptosis. Bax can destroy the outer membrane of mitochondria and cause the secretion of Cytochrome c. In contrast, as an anti-apoptosis protein, Bcl-2 can block Cytochrome c outflow and inhibit apoptosis (33). As the ratio of Bax/Bcl-2 protein is critical for cell survival, we explored the expression of Bax and Bcl-2 proteins after treating cells with SVPS2 for 24 h. The results showed Bax was up-regulated in a concentration dependent manner under SVPS2 treatment. Meanwhile, Bcl-2 was significantly down-regulated (Figure 10). Besides, the release of Cytochrome c promoted the activation of Caspase-9 and Caspase-3. Continuous activation of caspases is a critical step in the execution phase of apoptosis, therefore, the significantly increased expression levels of cleaved caspase-3 and caspase-9 confirmed the apoptosis of HT-29 cells. The presented data indicated that SVPS2 induced Bax translocation to the mitochondria and let off Cytochrome c, resulting the stimulation of the downstream signaling molecule. Many heteropolysaccharides demonstrated excellent immune-enhancing and anticancer activities. For instance, a heteropolysaccharide (BP-1) obtained from highland barley, comprising glucose, arabinose, xylose, and rhamnose in a molar ratio 8.82:1.50:1.92:1.00, induced HT-29 cells' apoptosis by improving the ratio of Bax/ Bcl-2 protein (34).

### The possible molecular mechanism of SVPS2

The apoptosis of tumor cells is generally triggered by the co-action of multiple apoptotic genes. It can manipulate the intrinsic pathway of apoptosis, in which death receptors transmit death signals to the mitochondria, leading to the permeabilization of the outer membrane. The release of Cytochrome c is mediated by the Bcl-2 family of proteins. Our results evidenced that SVPS2 could promote apoptosis by increasing the Cytochrome c release, translocation of Bax from the cytosol to mitochondria, activation of caspase-3 and caspase-9.

It's well-known that the function of anticancer was associated with a durable tumor-targeting immune response. As one type of important membrane receptors involved in activated macrophage, TLRs recognize the delivery by binding polysaccharides and then activates TNF receptor associated signal pathways including MAPKs and NF-κB downstream. Meanwhile, some proinflammatory (IL-6, IL-1β, TNF-α) would be released into the cytoplasm and elicited secondary immune responses following the activation of the immune signaling pathways. Similar phenomenon can be found in CMDP-4b, a polysaccharide obtained from *Cucurbita moschata Duch*, the immunomodulatory activity of it was executed by promoting the secretion of NO and cytokines (35).

In conclusion, the cultivation of *S. vaninii* in a forest environment has achieved successful application with the fast-growing techniques. It would not only avoid the cost of artificial greenhouses, but make a complete use of the natural shade conditions and the differences in day and night temperatures in a forest, thereby improve the biosysthesis of some high valuable biomacromolecules during the process of wild cultivation. In this study, a novel water-soluble β-galactoglucofurannan (SVPS2) was acquired from the cultivated mushroom of *S. vaninii* under forest. According to the analysis of structure, it demonstrated that the primary structure of SVPS2 was identified to consist of (1 → 5)-linked β-Glc*f* main chain with minor galactose and fucose on the branch. Its advanced conformation was further determined to be curly molecules in saline solution with approximate 0.65 nm height on average. The immunostimulation test indicated SVPS2 could facilitate the initiation of the immune reaction and promote the secretion of cytokines *in vitro*. SVPS2 could prevent the apoptosis of HT-29 cells into S phase. Moreover, SVPS2 could remarkably release some key proteins including Bax, Cytochrome c, cleaved caspase-3 and down-regulate the level of Bcl-2. Therefore, SVPS2 may be developed for the application on antitumor drugs and healthy food. The mechanism should be further verified with animal experiments.

## Data availability statement

The original contributions presented in the study are included in the article/Supplementary material, further inquiries can be directed to the corresponding author.

## Author contributions

JC: methodology, investigation, validation, formal analysis, data curation, and writing-original draft. YW: methodology, investigation, and visualization. JS: resources, methodology, and investigation. YL: investigation, data curation, validation, and visualization. WJ: resources, supervision, and investigation. LH: conceptualization, supervision methodology, writing-review and editing, project administration, and funding acquisition. HW: methodology, visualization, supervision, and funding acquisition. CH: methodology, software, and investigation. YJ, YX, and QH: methodology and investigation. XH: formal analysis. HD: methodology and validation. All authors contributed to the article and approved the submitted version.
