# Supplementary figures and images for: Characterization, immunostimulatory and antitumor activities of a β-galactoglucofurannan from cultivated Sanghuangporus vaninii under forest
Source: Front Nutr. 2022 Dec 22;9:1058131. doi: 10.3389/fnut.2022.1058131 (PMC9812957; doi:10.3389/fnut.2022.1058131)

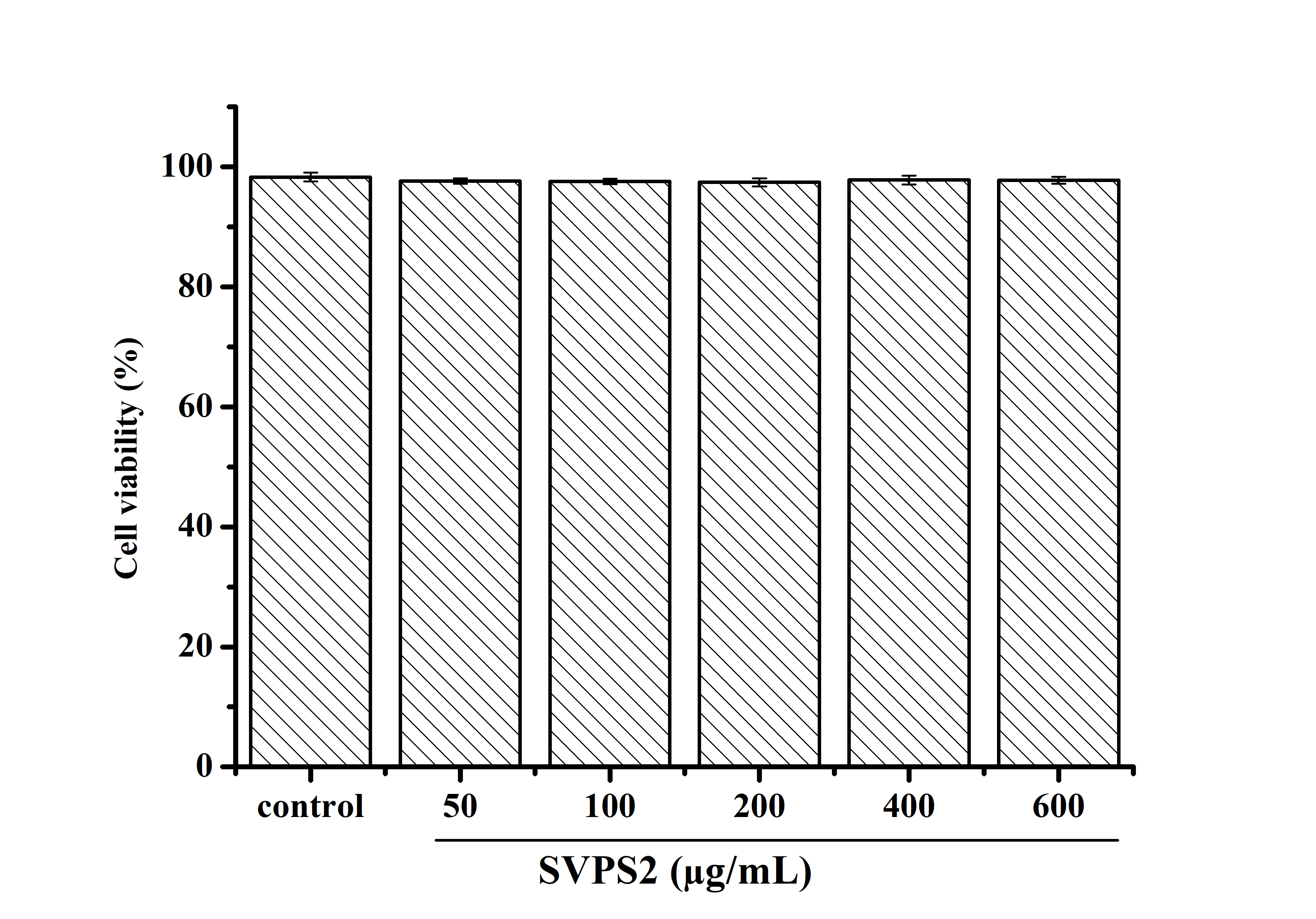

Supplement: Supplementary file 1 [file Image_1.jpg]
